# Contribution of polymorphisms in genes associated with craniofacial development to the risk of nonsyndromic cleft lip and/or palate in the Brazilian population

**DOI:** 10.4317/medoral.18357

**Published:** 2013-03-25

**Authors:** Lívia M. Paranaíba, Sibele N. de Aquino, Andreia Bufalino, Hercílio Martelli-Júnior, Edgard Graner, Luciano A. Brito, Maria R. Passos-Bueno, Ricardo-D. Coletta, Mário S. Swerts

**Affiliations:** 1 Professor, Stomatology Clinic, Dental School, State University of Montes Claros, Montes Claros, Minas Gerais, Brazil; 2 Pos Graduate, Department of Oral Diagnosis, School of Dentistry, State University of Campinas, Piracicaba, São Paulo, Brazil; 3 Professor, Department of Oral Diagnosis, School of Dentistry, State University of Campinas, Piracicaba, São Paulo, Brazil; 4 Pos Graduate, Human Genome Research Center, Institute of Biosciences, University of São Paulo, São Paulo, Brazil; 5 Professor, Human Genome Research Center, Institute of Biosciences, University of São Paulo, São Paulo, Brazil; 6 Professor, Center for Rehabilitation of Craniofacial Anomalies, Dental School, University of Alfenas, Alfenas, Minas Gerais, Brazil

## Abstract

Background and Objective: Nonsyndromic cleft lip and/or palate (NSCL/P) is a complex disease associated with both genetic and environmental factors. One strategy for identifying of possible NSCL/P genetic causes is to evaluate polymorphic variants in genes involved in the craniofacial development. Design: We carried out a case-control analysis of 13 single nucleotide polymorphisms in 9 genes related to craniofacial development, including TBX1, PVRL1, MID1, RUNX2, TP63, TGF?3, MSX1, MYH9 and JAG2, in 367 patients with NSCL/P and 413 unaffected controls from Brazil to determine their association with NSCL/P. Results: Four out of 13 polymorphisms (rs28649236 and rs4819522 of TBX1, rs7940667 of PVRL1 and rs1057744 of JAG2) were presented in our population. Comparisons of allele and genotype frequencies revealed that the G variant allele and the AG/GG genotypes of TBX1 rs28649236 occurred in a frequency significantly higher in controls than in the NSCL/P group (OR: 0.41; 95% CI: 0.25-0.67; p=0.0002). The frequencies of rs4819522, rs7940667 and rs1057744 minor alleles and genotypes were similar between control and NSCL/P group, without significant differences. No significant associations among cleft types and polymorphisms were observed. Conclusion: The study suggests for the first time evidences to an association of the G allele of TBX1 rs28649236 polymorphism and NSCL/P.

** Key words:**Cleft lip, cleft palate, polymorphism, genetic.

## Introduction

Cleft lip and/or palate (CL/P) is the most common congenital anomaly of the face, and its etiology, which involves both genetic and environmental factors, is highly complex and the molecular basis remains largely unknown (1,2). CL/P is frequently an isolated disorder, named nonsyndromic (NSCL/P, MIM 119530), but in some cases it is associated with other alterations characterizing a syndrome (2). It has been reported more than 300 syndromes with CL/P as a common feature (1,2). NSCL/P has a wide geographical distribution, with an average birth prevalence of approximately 1:700 (1). One strategy that has helped in the identification of genetic risks of NSCL/P is the evaluation of single nucleotide polymorphisms in genes responsible for craniofacial development.

In this study we investigated the contribution of selected polymorphic variants in genes related to craniofacial development as risk factors of NSCL/P. We selected the following genes: TP63, which is expressed in the epithelial cells during lip and palate formation and mutations are related to ectrodactyly, ectodermal dysplasia and cleft lip/palate syndrome (MIM 604292) (3), MID1 that is responsible for Opitz/GBBB syndrome (MIM 300000), a congenital disorder affecting primarily midline structures such as lip and palate (4), PVRL1 that encodes a cell-to-cell adhesion molecule (nectin1) expressed in the palatal shaves and related to cleft lip/palate-ectodermal dysplasia (MIM 225060) (5), RUNX2, which is largely expressed in the osteogenic mesenchyme during palate development (6) and that is responsible for cleidocranial dysplasia (MIM 119600), TBX1 that regulates cell adhesion and palatal development and is associated with 22q11.2 deletion syndrome (MIM 611867) (7), TGF?3, which shows pleiotropic biological effects and is known to induce palatal fusion (8), MSX1 that, based on mouse expression studies and knockout model, is considered a candidate for orofacial clefting and gene dosage in humans increases the risk for both orofacial clefting and oligodontia (9), MYH9, which has its encoded product abundantly and specifically expressed in epithelial cells of palatal shelves before fusion (10), and JAG2 that encodes a cell surface protein essential for palate development to prevent premature palatal shelf adhesion to other oral tissues and to facilitate normal adhesion between the elevated palatal shelves (11). Except for the polymorphisms rs7940667 in PVRL1 gene and rs1057744 in JAG2 gene, which have yielded inconsistent results (12-15) there are no previous studies evaluating the selected polymorphisms in NSCL/P patients.

## Material and Methods

-Samples

This study included 367 unrelated patients with NSCL/P recruited from the Center for Rehabilitation of Craniofacial Anomalies, Dental School, University of Alfenas, Brazil. All patients were carefully examined and screened for the presence of associated anomalies or syndromes by the team of the Center for Rehabilitation of Craniofacial Anomalies. The clefts were classified with the incisive foramen as reference, and divided in 3 groups: cleft lip only (CLO), cleft lip and palate (CLP), and cleft palate only (CPO). Controls were chosen among subjects admitted as inpatients in the Dental School of the same University with conditions unrelated to clefting disorders (n=413). The control group was matched by age, place of birth and ancestrality. In a recent study we characterized the ancestrality of those samples by using a panel of 40 insertion-deletion markers (16). The average ancestry of the NSCL/P sample was estimated at 84.1% of European, 13.4% of African and 2.5% of Amerindian, whereas ancestrality of control samples was 89.0% of European, 8.1% of African and 2.9% of Amerindian for controls. Written informed consents were obtained from all participants, and the study was carried out with approval of the Human Research Ethics Committee of the University.

-Genotyping

Genomic DNA was extracted from oral mucosa cells as previously described (17). Criteria for the selection of polymorphisms were as follows: (a) located within the region of an exon, (b) classified as non-synonymous polymorphism, (c) positioned in a restriction site, and (d) located in a conserved region of the gene in which alterations could result in biological effects. Thirteen non-synonymous polymorphic sites in TP63 (rs34713855), PVRL1 (rs7940667), MID1 (rs55986608), RUNX2 (rs11498200), TBX1 (rs28649236, rs28939675 and rs4819522), TGF?3 (rs34019007 and rs4252315), MYH9 (rs11549909 and rs11549910), MSX1 (rs62636562) and JAG2 (rs1057744) were examined by polymerase chain reaction-restriction fragment length polymor-phism (PCR-RFLP) analysis. Primers, restriction enzymes and PCR-RFLP conditions are available on request. After incubation with restriction enzymes, the products were electrophoresed on 8% nondenaturing polyacrylamide gels containing 0.5 µg/ml of ethidium bromide. For all polymorphisms, repeated genotyping analyses were randomly performed in 10% of the samples.

-Statistical Analyses

The distribution of the genotypes was evaluated by Hardy-Weinberg equilibrium test, and the frequency distribution of alleles and genotypes under unrestricted and dominant genetic models was compared using cross-tabulation and standard chi-square test. In unrestricted analysis, it was assumed that all 3 genotypes have different disease odds, whereas in dominant model the heterozygote and rare homozygote have the same disease odds. To estimate the risk of each factor in the occurrence of NSCL/P, odds ratios (OR) and respective 95% confidence intervals (95% CI) were calculated. Multiple comparisons were corrected using the Bonferroni method. The assessment of gene-gene interactions was carried out using multifactor dimensionality reduction (MDR), which identifies combinations of genotypes associated with risk for the disease. In this study, we used 10-fold cross validation and 1000-fold permutation testing.

## Results

From 13 polymorphisms, only 4 of them, including rs28649236 and rs4819522 of *TBX1*, rs7940667 of *PVRL1* and rs1057744 of JAG2, demonstrated genotypic variations in our population. The genotype distributions were consistent with Hardy-Weinberg equilibrium at all loci except for rs28649236 in *TBX1*.

Allelic and genotypic frequencies are depicted in  Table 1, Table 2. The percentage of the variant allele of *TBX1* rs28649236 polymorphism was significantly lower in NSCL/P patients than that in controls (p=0.0002), with an odds ratio of 0.41 (95% CI: 0.25-0.67) ( Table 1). The frequency of the rare genotypes AG and GG were significantly higher in control group compared to NSCL/P group (p=0.01), but this significance did not remained after Bonferroni correction for multiple comparisons ( Table 2). However, when AG and GG were combined and compared against AA genotype (dominant genetic model), a significant association was observed. The AG/GG genotype was significantly more frequent in the control group (10.4%, n=43) than in the NSCL/P group (4.9%, n=18) (p=0.004), revealing an association with NSCL/P (OR: 0.44, 95% IC: 0.25-0.78). The frequency of *TBX1* rs4819522 genotypes in a dominant genetic model (CC vs CT+TT) was different between groups, but this difference was not kept after Bonferroni correction ( Table 2). Similarly, PVRL1 rs7940667 CC genotype was more frequent in the control group compared with NSCL/P group, but this difference did not resist Bonferroni adjustment ( Table 2). The frequency of the GA genotype for rs1057744 *JAG2* polymorphism was higher than GG and AA genotypes, but no significant differences between groups were observed ( Table 2). There were no significant associations, after Bonferroni adjustment, between polymorphisms and cleft types ( Table 3). However, our data indicated a marginal significance between *TBX1* polymorphisms and CLP and between PVRL1 rs7940667 polymorphism and CPO.

**Table 1:**
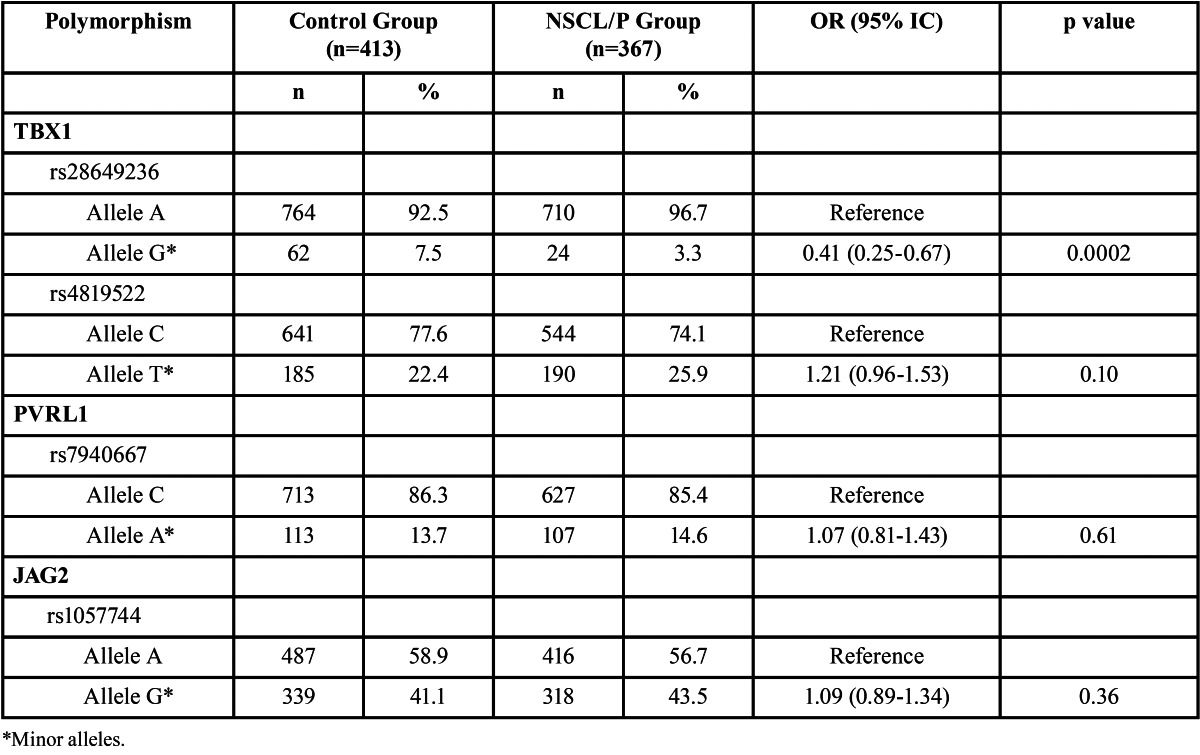
Allelic frequencies of *TBX1, PVRL1* and *JAG2* polymorphisms in the control and NSCL/P groups. Major alleles were used as reference for comparisons.

**Table 2:**
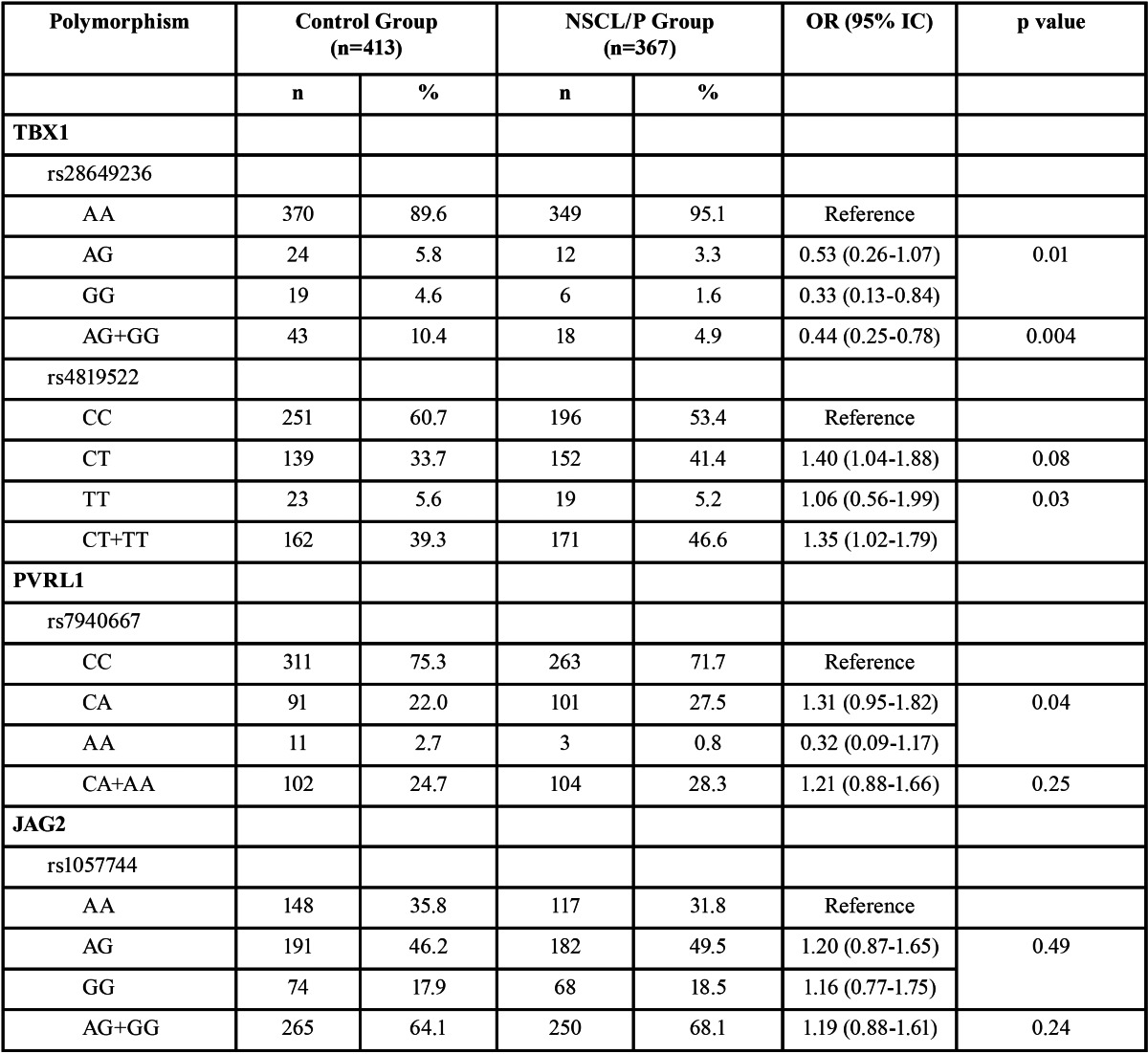
Genotype distributions under unrestricted and dominant genetic model of *TBX1, PVRL1* and *JAG2* polymorphisms in the control and NSCL/P groups.

**Table 3:**
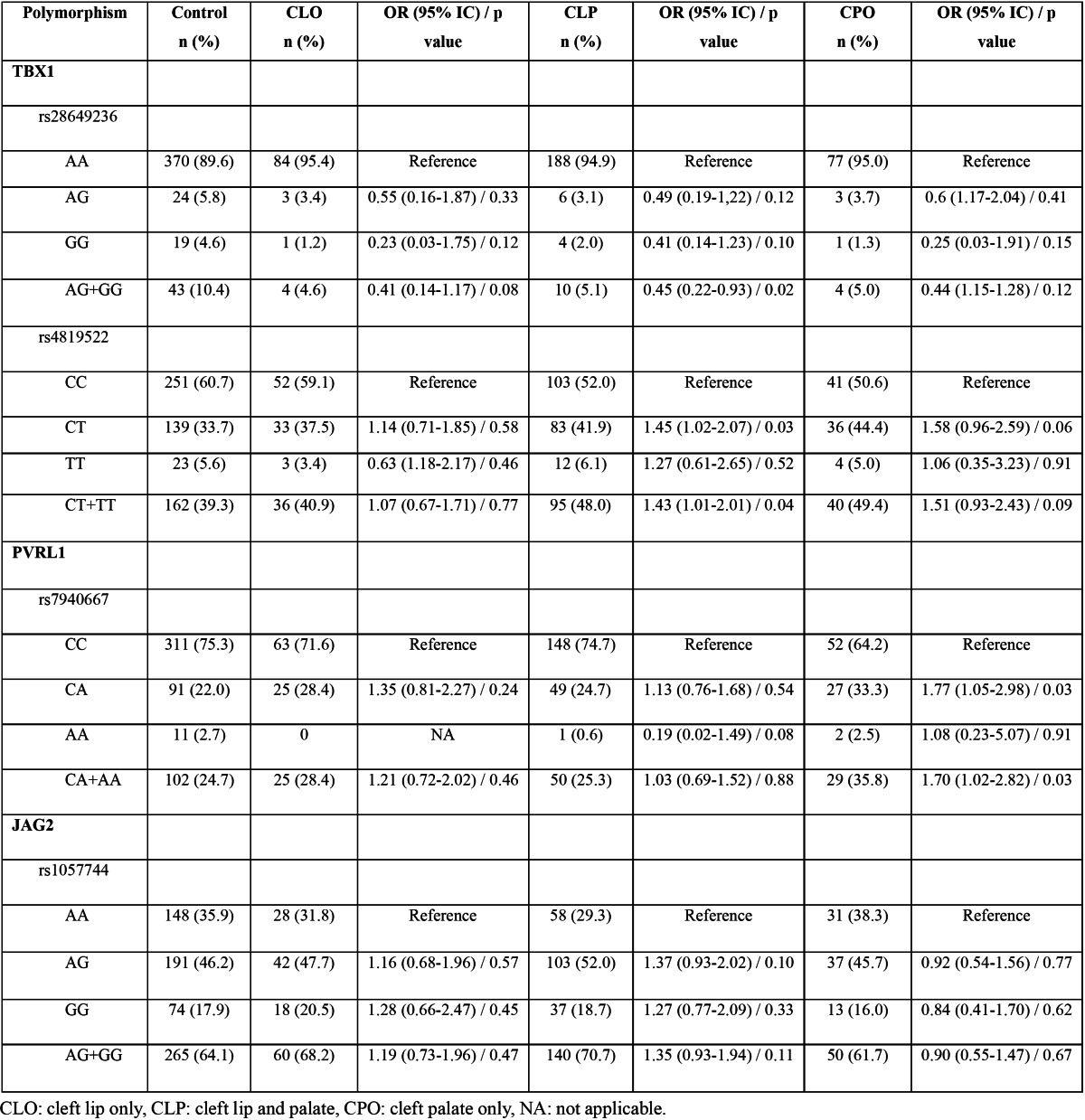
Genotype distributions of *TBX1, PVRL1* and *JAG2* polymorphisms by type of cleft. Control group was used for comparison.

Potential interactions among the genes were assessed using MDR analysis ( Table 4). The combination of *TBX1* rs28649236 and *PVRL1* rs7940667 was the best two factor model with a testing accuracy of 0.52. The best three factor combination, *TBX1* rs28649236, *PVRL1* rs7940667 and *TBX1* rs4819522, demonstrated the accuracy of 0.59 and the cross validation consistency of 7/10. However, no model was significant in predicting the individual risk for NSCL/P.

**Table 4:**
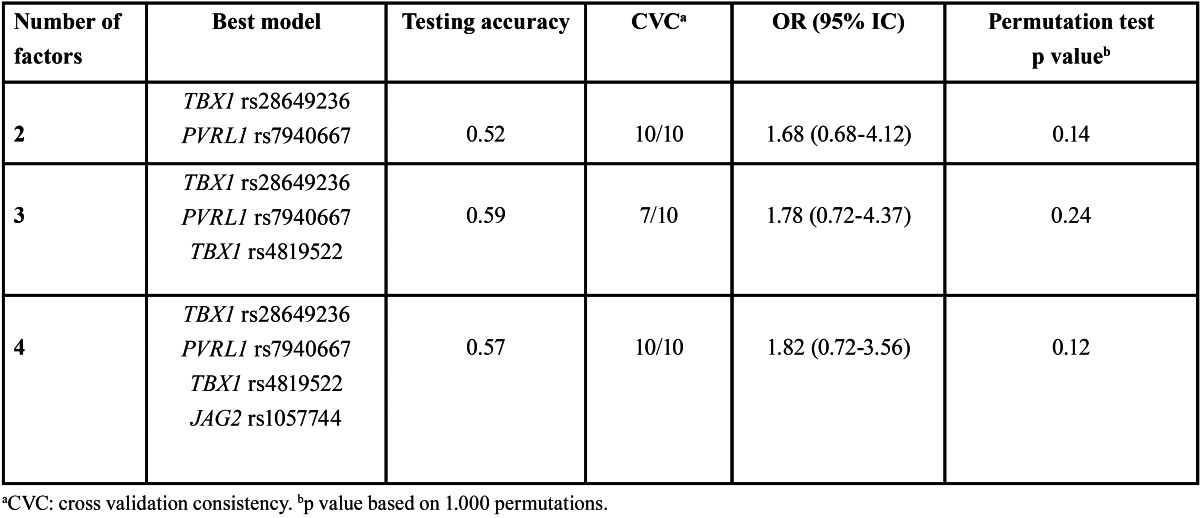
Interactions between *TBX1, PVRL1* and *JAG2* and risk prediction of NSCL/P by multifactor dimensionality reduction method.

## Discussion

Several evidences indicate that NSCL/P, as resulted of genetic heterogeneity, low penetrance and influence of a variety of environmental factors, is a genetically complex disease (1,2). Although there has been marked progress in identifying genetic and environmental triggers for NSCL/P, the etiology remains poorly characterized. In this study we evaluated 13 polymorphisms in 9 genes in patients with NSCL/P, and found that the G allele of TBX1 rs28649236 polymorphism was significantly associated with occurrence of NSCL/P, even after correction for multiple comparisons.

The rs28649236 nonsynonymous polymorphism in TBX1 replaces an arginine by a glycine at amino acid position 8 (p.Arg8Gly) of the protein sequence. Although there is no information of effect of this polymorphism on protein function, by using bioinformatic analysis (18) we observed that the polymorphic nucleotide is located in a putative area used for alternative splicing and the substitution may result in an alteration on the encoded protein, suggesting biological effects. The G variant allele was significantly more frequent in the control group than in the NSCL/P group, and an individual carrying the G allele exhibited a ~2.5-fold lower risk to having NSCL/P. However, the distribution of alleles and genotypes showed no association with the type of cleft. A statistical significance of the rs28649236 deviation from Hardy-Weinberg equilibrium was observed. This distortion may be due to genotyping errors or random chance, since 1 in every 20 markers can present imbalance (19). Although we have repeated genotyping analysis in more than 10% of the samples for quality control and the results were totally concordant, the possibilities of PCR genotyping errors because of nucleotide sequence mutations, unreported polymorphisms and INDELS at the PCR primer sites exist. In addition, previous studies with the Brazilian population also found Hardy-Weinberg imbalance, which was credited to the ethnic complexity of our population (20,21). Brazilians form one of the most heterogeneous populations in the world, which is the result of five centuries of interethnic crosses of peoples from three continents: the European colonizers, mainly represented by the Portuguese, the African slaves, and the Amerindians (22). Thus, the significance of the G allele of rs28649236 in TBX1 might be related to higher European contribution present in our sample. To date, there is no previous studies evaluating this polymorphism in NSCL/P. Han and colleagues (23) demonstrated an increased frequency of the G allele in children with conotruncal defects. TBX1 mRNA expression was described to be essential to coronary tissue development during embryogenesis (24), supporting the association of rs28649236 with heart defects. Interestingly, TBX1 was recently associated with the elongation and elevation of the palatal shelves during palatogenesis (25). Thus, this significant result provides evidence of a new susceptibility marker for NSCL/P, and supports future studies involving larger groups of patients and other ethnic populations.

Borderline associations with NSCL/P were found with other two markers of this study. We have found that CT and TT individuals for TBX1 rs4819522 polymorphism have an increased risk of NSCL/P in our population. However, this association did not resist the adjustment for multiple comparisons of Bonferroni. One of the main issues in association studies is how to evaluate the significance of multiple testing of polymorphims. Bonferroni correction is commonly applied, but it is too stringent, whereas an alternative approach would be to use replication of a nominal probability in a second data set, which is less rigid. Since we did not have access to a second dataset, we applied the Bonferroni correction. As significant associations for this study required an adjusted p?0.004, the significance of TBX1 rs4819522 polymorphism was lost. We realize that this correction is a limitation and may lead to loss of significant findings, but in light of not having verification, this approach appeared to be the most efficacious. No previous studies assessed the correlation of this polymorphism with NSCL/P. Because of the marginal association, this candidate marker requires replication in other sample sets, and also should impel others to investigate such association in different populations.

The first evidence in favor of a role for PVRL1 variants in NSCL/P was obtained by Sözen and collaborators (26). Those authors demonstrated a strong association between PVRL1 W185X mutation and NSCL/P in individuals from Venezuela. The same group demonstrated Pvrl1 mRNA expression in the medial edge epithelium of the palatal shelves (5). Allelic and genotypic association studies of PVRL1 polymorphisms and mutations in NSCL/P patients have yielded inconsistent results, but overall do support the association of PVRL1 defects with risk of NSCL/P (12-14,27,28). In particular, PVRL1 rs7940667 polymorphism was significantly associated with the development of NSCL/P, especially in the isolated forms of CL and CP, in populations from Iowa-USA, Philippines and Denmark (12). Recently, a lack of association between rs7940667 polymorphism and North American Caucasian, Australian and Guatemalan NSCL/P patients was reported (13,14). JAG2 encodes a ligand for Notch pathway receptors and is required for craniofacial, limb and T cell development (29). The first evidence showing that JAG2 may be related to oral clefting came from animal studies, where Jag2 knockout mice die at birth due to completely penetrant cleft palate (11). Later, several studies have evaluated the participation of JAG2 genetic variants in NSCL/P, but the results are inconsistent (13,15,30). As in the present study, a lack of association between rs1057744 polymorphism and NSCL/P was reported in single polymorphism analysis (18). However, a strongest association was observed in haplotype analyses containing this polymorphism (18). Therefore, further studies are required to explore the precise contribution of JAG2 variants and/or haplotypes in NSCL/P in the Brazilian population.

On examining epistatic interactions, our results showed no significant correlation between the combinations and the risk for NSCL/P. Thus, rs28649236 of TBX1 gene appears to contribute independently to a decreased NSCL/P risk. Finally, nine of the probably polymorphisms of the selected candidate genes were not confirmed in the Brazilian population. These results are not surprising since two of them (TP63 rs34713855, RUNX2 rs11498200, TGF?3 rs4252315, MSX1 rs62636562) are invariable in practically all populations studied (GenBank, http://www, ncbi.nlm.nih.gov), and the others (MID1 rs55986608, TBX1 rs28939675, MYH9 rs11549909 and rs11549910, TGF?3 rs34019007) have never been studied before.

In conclusion, this study suggests an association between the G allele of TBX1 rs28649236 polymorphism and the development of NSCL/P in the Brazilian population, while that the associations of TBX1 rs4819522, PVRL1 rs7940667 and JAG2 rs1057744 with NSCL/P require further studies.
